# The relationship between vision status and its influencing factors among primary and secondary school students: The masking effect of physical activity level and the moderating effect of academic stress

**DOI:** 10.1186/s12889-025-21625-7

**Published:** 2025-02-05

**Authors:** Jiyan Xu, Yanjun Li, Siyuan Ma, Shimeng Dai, Weiwei Xu, Mengjiao Sang, Kaijie Feng

**Affiliations:** 1 School of Recreation and Community Sport, Capital University of Physical Education and Sports, Beijing, China; 2 China Wushu School, Beijing Sport University, Beijing, China; 3 Physical Education Department, Beijing Information Science and Technology University, Beijing, China; 4 College of Humanities and Law, Beijing University of Chemical Technology, Beijing, China; 5 School of Pharmaceutical Sciences, Shandong University, Jinan, Shandong China; 6 Department of Physical Education and Research, Peking University, Beijing, China

**Keywords:** Lifestyle behaviors, Myopia, Physical activity level, Academic stress, Masking effect, Moderating effect

## Abstract

**Background:**

The global youth myopia rate continues to rise, and the vision health of primary and secondary school students has become a global concern. This cross-sectional study aims to explore the vision status of primary and secondary school students in the Ningxia Hui Autonomous Region, China, and its internal relationship with influencing factors under regional characteristics.

**Methods:**

A survey was conducted among 1,670 primary and secondary school students in the Ningxia Hui Autonomous Region of China using a snapshot method and random sampling techniques from September to October 2023. The survey instruments included vision examination, the International Physical Activity Questionnaire, the Lifestyle Behavior Scale, and the Student Academic Stress Questionnaire. The data was tested with descriptive statistics, difference test, correlation analyses, regression analyses, and so on using SPSS 25.0 and SPSSAU, and the target model was established and tested for the goodness-of-fit with AMOS 23.0.

**Results:**

There was a significant negative correlation between vision status and lifestyle behaviors (*r*=-0.360, *p* < 0.01), physical activity level (*r*=-0.058, *p* < 0.05). The physical activity level played a significant masking effect between lifestyle behaviors and vision status (a*b = 0.002, c’=-0.044), and academic stress played a moderating role in the process of lifestyle behaviors affecting physical activity level and vision status. A moderated mediator model was constructed and fitted well (RMSEA = 0.028, CFI = 0.953, NFI = 0.934).

**Conclusions:**

The prevalence rate of myopia among primary and secondary school students in Ningxia Hui Autonomous Region is lower than the national average, and its prevalence is related to lifestyle behaviors, physical activity level, and academic stress. The impact of lifestyle behaviors on vision status is controlled by physical activity level and academic stress. A strategy of coordinated education involving families, schools, and communities should be implemented. Parents should avoid having excessive expectations and additional academic burdens on their children and encourage them to actively participate in sports; schools should fully implement the ‘’Double Reduction’’ policy, making myopia prevention and control an important part of health education; communities should carry out publicity work for myopia prevention and control, raising adolescents’ awareness of eye health.

## Introduction

Nearsightedness is a refraction anomaly where external parallel light rays focus in front of the retina after passing through the eye’s optical system [1]. In recent years, the global prevalence of myopia among adolescents has been steadily increasing, making the visual health issues of primary and secondary school students a focus of global attention. In October 2019, the World Health Organization released the “World Report on Vision”, indicating that at least 2.6 billion people worldwide suffer from visual impairment or blindness, with myopia becoming one of the most prevalent eye disorders. The report also highlighted the alarming situation of myopia prevalence among Chinese adolescents [2]. Specifically, the myopia rates among primary school students, junior high school students, and senior high school students are 35.6%, 71.1%, and 80.5% respectively, showing a trend towards younger age groups. Visual impairment can have adverse effects on the physical and mental development, quality of life, and learning outcomes of children and adolescents, while also increasing the risk of high myopia and related vision disorders in adulthood [3].

There are numerous factors influencing the visual health of adolescents, and the related theories and mechanisms are complex [4]. Among them, lifestyle behaviors, physical activity level, and academic stress all have an impact on the visual status of young individuals [5–6]. Lifestyle behaviors are important factors affecting health and also play a crucial role in influencing the visual health of adolescents [5]. Lifestyle behaviors, including eye use, exercise, and sleep habits, directly impact the vision status of adolescents. Physical activity refers to any activity that causes skeletal muscle contraction and increases energy consumption beyond the resting level [7], including sports, recreational activities, and household chores. Some studies suggest a relationship between physical activity level and myopia [8]. Mutti et al., through a 5-year longitudinal study, found a negative correlation between physical activity level and myopia in children [9], A surge of observational studies on physical activity and myopia followed, primarily exploring the correlation between physical activity level and myopia [10–12]. Since lifestyle behaviors also include exercise, lifestyle behaviors may to some extent influence physical activity level, suggesting a correlation between lifestyle behaviors and physical activity level, as well as their relationship with myopia. Pressure can be divided into stressors and stress responses, with academic stress referring to environmental demands and challenges in the academic environment that exceed the resources available to students [13]. This study selected academic stress as the main variable affecting visual health for empirical research. The study found that academic stress increases study and homework time, reduces students’ sleep and exercise time, subsequently increasing the prevalence of myopia and impacting students’ visual health [14]. Students’ academic stress is significantly negatively correlated with sleep and exercise time, and positively correlated with myopia rates. Schools and families that focus excessively on academic achievement, sacrificing students’ exercise and sleep time to extend study hours, increase students’ academic burden, thereby affecting their visual health adversely [15]. Therefore, it is inferred that academic stress affects lifestyle behaviors and physical activity level, consequently impacting students’ vision status.

Based on the aforementioned analysis, this paper presents three hypotheses: (1) The lifestyle behaviors of primary and secondary school students can significantly predict myopia; (2) The physical activity level of primary and secondary school students plays a mediating role between lifestyle behaviors and myopia; (3) Academic stress of primary and secondary school students plays a moderating role between lifestyle behaviors and myopia. Based on these hypotheses, the relationship between the lifestyle behaviors, physical activity level, academic stress, and vision status of primary and secondary school students forms a moderated mediation model. Studying the mediating role can elucidate how lifestyle behaviors affect myopia, studying the moderating role can reveal the extent of the impact of lifestyle behaviors on myopia, and studying the moderated mediation effect can make the factors influencing visual health more specific and mechanisms clearer. The Ningxia Hui Autonomous Region, as a multi-ethnic settlement area, may see its diverse cultural background and unique geographical environment exert certain influences on the visual health of adolescents. Taking the Ningxia Hui Autonomous Region in China as an example, this paper explores the occurrence of myopia among primary and secondary school students in the context of regional characteristics and its relationship with lifestyle behaviors, physical activity level, and academic stress. It aims to provide guiding suggestions for myopia prevention in this region and advance efforts in myopia prevention among primary and secondary school students.

## Research design

### Objects of the study

The research focuses on the prevalence of myopia among primary and secondary school students in the Ningxia Hui Autonomous Region in China and its influencing factors. The survey was conducted online using the Wenjuanxing online survey platform from September 28 to October 12, 2023. Before administering the survey, the instructions were explained to the participants by the responsible person or teacher, informing them of the anonymity, confidentiality, and purpose of the survey. Participants completed the questionnaire voluntarily after agreeing to participate. Demographic information such as gender (male = 1, female = 0), age, and grade level (primary school = 0, secondary school = 1) was collected during the survey, with 1,670 valid questionnaires collected. Among them, 50.3% were male and 49.7% were female; 81.4% were in primary school and 18.6% were in secondary school. Written informed consent was obtained from the legal guardians of all the participants and consent was also obtained from every participant. The data collected were quantified using Excel. Due to the impact of missing data on the results of this study, the missing values were removed. Descriptive statistics, correlation and regression analyses, as well as moderation effect tests were conducted using SPSS 25.0, SPSSAU, and AMOS 23.0. The Bootstrap method was employed to verify the mediating effect of the physical activity level, and the hypothesis model was tested using AMOS 23.0.

### Research method

#### Vision examination

The research team for this study included at least one ophthalmologist licensed as a national practitioner in ophthalmology, with expertise in optometry, and several professionals holding certifications as technicians or nurses in vision-related fields. Two types of tests were used for the visual acuity assessment. One is the use of a standard logarithmic visual acuity meter in accordance with the national standard (GB 11533), which is placed 5 m in front of the examined eye, and before the test, the requirements and purposes are made clear, and the subject is asked to speak or make the corresponding gestures to indicate the direction of the visual standard gap [16]. Secondly, a computerized optometry instrument conforming to the pharmaceutical industry standard (YY 0673–2008) was used, and under the condition of non-ciliary muscle paralysis, each eye of the examinee was measured three times and the average value was taken [17]. The criteria for diagnosing myopia were based on the following standard: uncorrected visual acuity < 5.0 and equivalent spherical power under non-cycloplegic conditions < -0.50 diopters cylinder. Prevalent conditions such as strabismus, pathological high myopia and retinal disease were excluded prior to the study.

#### International physical activity questionnaire-short form

The International Physical Activity Questionnaire-Short Form (IPAQ-SF) was used to measure the weekly frequency and daily cumulative time of different intensity activities of the survey subjects, with a total of 7 items. The first 6 items inquired about the physical activity of the subjects, and the 7th item assessed the sedentary time of the subjects. Walking was assigned a Metabolic Equivalent Task (MET) value of 3.5, moderate-intensity activity 4.5, and high-intensity activity 8.0.

The study utilized physical activity grouping variables as indicators to assess the level of physical activity. Following previous research [18], physical activity was divided into low, moderate, and high groups (refer to Table 1). The measured sample characteristic value distribution in this study was 1.55 ± 0.79 (M ± SD), and Cronbach’s α was 0.791.

**Table 1:** Physical activity grouping criteria

Group	Standard
Low	Meet any 1 of the following 2 criteria: a. No physical activity; b. Physically active but not meeting intermediate or advanced standards;
Medium	Satisfy any 1 of the following 3 criteria: a. High-intensity activity at least 3 days/week, averaging at least 20 min per day; b. Moderate-intensity activity at least 5 days/week, averaging at least 30 min per day; c. A mixture of the 3 intensities for at least 5 days/week and a total weekly gross force activity level of at least 600 MET* minutes/week;
High	Satisfy any 1 of the following 2 criteria: a. High-intensity activity at least 3 days/week with a total weekly gross force activity level of at least 1500 MET* minutes/week; b. A mix of the 3 intensities for at least 7 days/week and a total weekly gross force activity level of at least 3000 MET* minutes/week.

#### Lifestyle behavior scale

The Lifestyle Behavior Scale consists of four scales: sleep behavior, eating behavior, eye behavior, and sports behavior, with a total of 18 items, distributed as 4, 3, 8, and 3 questions respectively, the full marks for the four dimensions of the theory are 10 points, 13 points, 31 points, and 5 points, respectively [19]. There are two types of questions: binary type using “yes (1 point) or no (0 point)” and Likert scoring method with options of 3 and 5, scored positively from “1 to 3” and “1 to 5” respectively. The theoretical total score of the scale is 58 points, and quantitative data were analyzed in this study. After forming the scale, content validity was assessed by experts, and after meeting the requirements, the structural validity and reliability of the scale were tested. In the first round, 144 exploratory questionnaires were distributed and collected, and exploratory factor analysis was used to test the structural validity, which was deemed qualified. In the second round (*n* = 1,670), the results were shown in Table 2, where all items met the requirements. The absolute skewness values of each item ranged from 0.453 to 1.367, kurtosis absolute values ranged from 0.095 to 1.624, and the minimum standard deviation was 0.774. The Kolmogorov-Smirnov normality test (K-S) normality test was significant (*P* < 0.001). The Cronbach’s α of the scale was 0.686, split-half reliability was 0.727, and item-total correlations ranged from 0.289 to 0.514 (*P* < 0.01, see Table 2), indicating good reliability of the scale [20].

#### Student stressor scale

The questionnaire used in this study was the revised Student Stressor Scale (SSS) by Chen Xu, comprising nine dimensions: task pressure, time pressure, demand pressure, competitive pressure, setback pressure, environmental pressure, pressure from others’ expectations, pressure of achievement goals, and pressure for self-development, totaling 62 items [21]. The scale employed a Likert 5-point scoring method with positive scoring. The theoretical total score of the questionnaire was 310 points, divided into five levels: “no pressure (0 ≤ SSS ≤ 62), a little pressure (62 < SSS ≤ 124), moderate pressure (124 < SSS ≤ 186), high pressure (186 < SSS ≤ 248), and extremely high pressure (248 < SSS ≤ 310).” The measured sample characteristic value distribution in this study was 146.78 ± 57.99 (M ± SD), with a Cronbach’s α of 0.993, meeting the reliability requirements (Table 2).

**Table 2:** Exploratory factor analysis and validated factor analysis indicators of lifestyle behaviors scale and SSS

Scale	Exploratory factor analysis				Validation factor analysis							
	KMO	Bartlett-test	df	*P*	χ2/df	GFI	NFI	IFI	NNFI	CFI	RMSEA	SRMR
Lifestyle behaviors	0.709	5095.411	153	< 0.001	7.093	0.955	0.903	0.914	0.907	0.912	0.054	0.046
SSS	0.993	184293.728	1491	< 0.001	6.672	0.942	0.915	0.923	0.919	0.923	0.061	0.024

## Result

### Common method bias

Using the Harman single-factor test to examine common method bias, the results indicated that the first factor explained 32.87% of the total variance, which met the criteria for judgment [22], indicating that there was no serious common method bias among the study variables.

### Overall characterization of myopia, lifestyle behaviors, physical activity level, and academic stress

As shown in Table 3, the proportion of myopic students is 27.3%, while students with normal vision account for 72.7%. Among students with different levels of physical activity, 64.2% are at a low level, 16.8% are at a moderate level, and 19.0% are at a high level. Independent sample t-tests and chi-square tests revealed significant gender differences in vision status, physical activity level, and academic stress. Female students have a higher myopia rate and academic stress than male students but a lower level of physical activity. There are no significant gender differences in lifestyle behaviors. Significant differences were also found across different academic stages in vision status, physical activity level, academic stress, and lifestyle behaviors. The myopia rate, physical activity level, and academic stress are higher among junior high school students compared to primary school students, but lifestyle behaviors scores are lower.

**Table 3:** Group differences in myopia or not, physical activity level, academic stress, and lifestyle behaviors

Category		Myopia		Physical activity level	Academic stress	Lifestyle behaviors	Lifestyle behaviors			
		Yes	No				Sleep behavior	Eating behavior	Sports behavior	Eye behavior
Gender	Male (*n* = 840)	23.4%	76.6%	1.61 ± 0.84	2.69 ± 0.99	47.01 ± 3.77	8.07 ± 1.27	9.44 ± 1.90	4.59 ± 0.68	25.05 ± 2.57
	Female (*n* = 830)	31.5%	68.5%	1.49 ± 0.74	2.78 ± 0.95	47.03 ± 3.87	8.03 ± 1.32	9.50 ± 1.98	4.45 ± 0.79	25.02 ± 2.57
	*t*/*χ2*	13.823		-3.079	2.047	0.065	-0.538	0.640	-4.057	-0.206
	*P*	< 0.001		< 0.001	0.026	0.130	0.213	0.251	< 0.001	0.645
	Cohen’s d	-0.179		0.151	-0.092	-0.005	0.031	-0.031	0.189	0.011
Segments	Primary school (*n* = 1360)	22.3%	77.7%	1.50 ± 0.78	2.65 ± 0.97	47.48 ± 3.59	8.13 ± 1.29	9.54 ± 1.89	4.57 ± 0.69	25.27 ± 2.48
	Secondary schools (*n* = 310)	49.7%	50.3%	1.75 ± 0.83	3.11 ± 0.89	45.00 ± 4.11	7.71 ± 1.28	9.15 ± 2.13	4.29 ± 0.85	23.99 ± 2.72
	*t/χ2*	95.342		-4.856	-7.564	10.671	5.131	3.186	6.094	8.087
	*P*	< 0.001		0.005	< 0.001	0.004	0.934	0.004	< 0.001	0.258
	Cohen’s d	-0.606		-0.311	-0.494	0.643	0.327	0.245	0.362	0.492

### The impact of physical activity level, academic stress, and lifestyle behaviors on myopia

As shown in Table 4, through Spearman correlation analysis, it was found that lifestyle behaviors and physical activity level were significantly negatively correlated with academic stress (*r*=-0.294, *P* < 0.01; *r*=-0.051, *P* < 0.05) and myopia status (*r*=-0.360, *P* < 0.01; *r*=-0.058, *P* < 0.05). Additionally, lifestyle behaviors and physical activity level were significantly negatively correlated (*r*=-0.125, *P* < 0.01), while academic stress was positively correlated with myopia status (*r* = 0.136, *P* < 0.01).

**Table 4:** Correlation analysis of lifestyle behaviors, physical activity level, academic stress and myopia

Variable	M	SD	1	2	3	4
1.Myopia	0.27	0.45	1			
2.Lifestyle behaviors	47.02	3.82	-0.360**	1		
3.Physical activity level	1.55	0.79	-0.058*	-0.125**	1	
4.Academic stress	2.74	0.97	0.136**	-0.294**	-0.051*	1

(Notes: *P < 0.05, **P < 0.01, the same as below.)

### Mediating effect test

This study employed the Bootstrap estimation method to randomly resample 1,670 primary and secondary school students, estimating the 95% confidence interval for the mediation effect. The results revealed that the indirect effect of physical activity level on myopia through lifestyle behaviors was 0.002, with a 95% confidence interval of [0.006, 0.021]. At this point, both coefficients a and b were significant. The opposite signs between the mediation effect (0.002) and the direct effect (-0.044) indicate that the physical activity level of primary and secondary school students plays a masking effect in the process of lifestyle behaviors influencing myopia (Table 5) [23].

**Table 5:** Results of the masking effect test

Term	Total effect	a	b	Intermediary effect	SE	z	*P*	a*b (95% BootCI)	Direct effect	Inspection conclusion
Lifestyle behaviors→Physical activity level→Myopia	-0.042**	-0.026**	-0.059**	0.002	0.004	0.403	0.006	0.006~0.021	-0.044**	Masking effect

### Moderating effect test

Models 1 to 2 used physical activity level as the dependent variable, while models 3 to 5 used myopia as the dependent variable, employing binary logistic regression analysis. The Variance Inflation Factor (VIF) values in the models were all less than 5, indicating no collinearity issues. As shown in Table 6, in Model 1, lifestyle behaviors (X) had a significant negative effect on physical activity level (W), and after adding academic stress and the interaction term between academic stress and lifestyle behaviors, all variable coefficients remained significant. In Model 3, both lifestyle behaviors (X) and physical activity level (W) had significant negative effects on myopia (Y), and after including academic stress and the interaction term between academic stress and lifestyle behaviors, all variable coefficients remained significant. This suggests that academic stress plays a moderating role in the process where lifestyle behaviors influence physical activity level and myopia [24]. In addition, since Model 1 only had one independent variable, a collinearity test was not conducted. Moreover, for Models 2 to 5, the Tolerance values for all variables were less than 1, and the Variance Inflation Factor (VIF) values were between 1 and 2, indicating that there were no significant collinearity issues among the variables.

**Table 6:** Results of logistics regression analysis

	Physical activity level		Myopia		
	Model 1	Model 2	Model 3	Model 4	Model 5
Constant	2.768**	2.963**	11.380**	10.642**	7.137**
Lifestyle behaviors(X)	-0.026**	-0.030*	-0.253**	-0.245**	-0.170**
Physical activity level(W)			-0.389**	-0.389**	-0.385**
Academic stress(U)		-0.075*		0.121*	1.346*
X×U		0.002*			-0.026*
R^2^	0.016	0.016	0.138	0.140	0.141
ΔR^2^	0.015	0.014	0.200	0.202	0.204

**Fig. 1:**
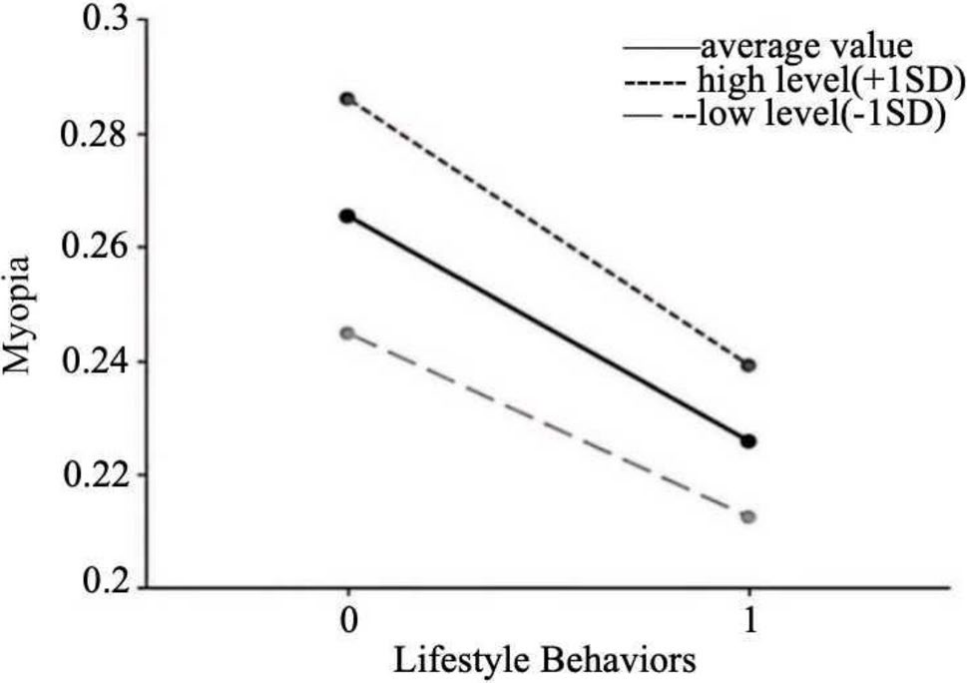
The moderating role of academic stress in the relationship between lifestyle behaviors and myopia

**Fig. 2:**
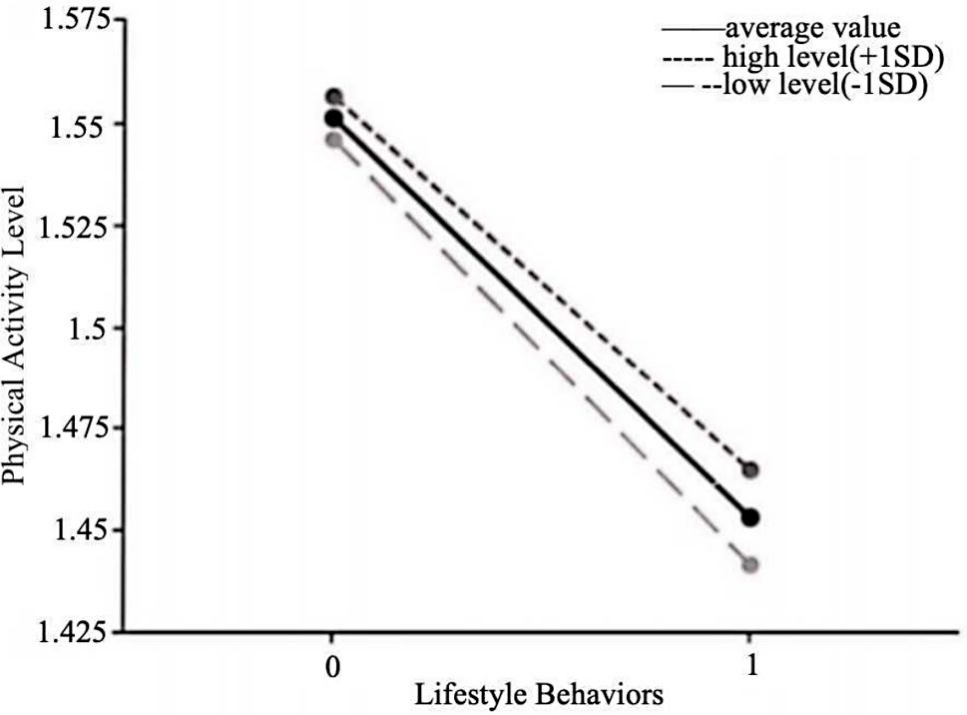
The moderating role of academic stress between lifestyle behaviors and physical activity level

To further explore the moderating effect of academic stress, this study employed simple slope analysis. Academic stress was taken at its mean value and mean value plus or minus one standard deviation, and simple effect graphs of academic stress on the relationship between lifestyle behaviors and myopia were plotted (Fig. 1). The analysis revealed that when academic stress was high (β=-0.047, *P* < 0.001), the impact of lifestyle behaviors on myopia was significantly stronger than when academic stress was low (β=-0.032, *P* < 0.001). Similarly, simple effect graphs of academic stress on the relationship between lifestyle behaviors and physical activity level were plotted (Fig. 2). The analysis found that when academic stress was low (β=-0.104, *P* < 0.001), the effect of lifestyle behaviors on physical activity level was significantly stronger compared to when academic stress was high (β=-0.092, *P* < 0.001).

### Relationship model of physical activity level, academic stress, lifestyle behaviors, and myopia

**Fig. 3:**
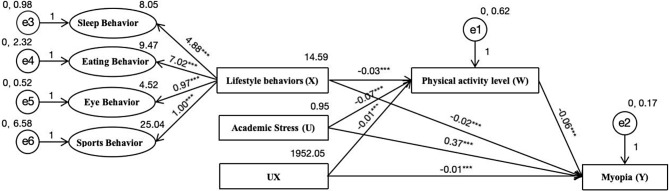
Moderated mediation models

Using AMOS 23.0 software, the impact paths of the structural equation model were analyzed (Fig. 3). Considering that the chi-square value increases with sample size, leading to rejection of any model [25], this study does not use the chi-square value as the basis for judging model fit. The absolute values of the CR values for all paths in the model were greater than 1.96, indicating that the significance of these parameters exceeded the 95% confidence level. The SE values for all paths were less than 1, reaching the statistical significance criterion (*p* < 0.05). The model indicates that lifestyle behaviors, academic stress, and the interaction term between academic stress and lifestyle behaviors all have significant negative impacts on physical activity level. Lifestyle behaviors and physical activity level negatively affect myopia, while academic stress positively influences myopia. In this model, physical activity level and academic stress play a masking effect and a moderating effect, respectively, jointly constituting a mediated moderation model. The model’s Root Mean Square Error of Approximation (RMSEA, it is an indicator for measuring model fit, where a lower value indicates better model fit, generally it should be less than 0.08.) = 0.028 < 0.05, Comparative Fit Index (CFI, it is an indicator of comparative fit, ranging from 0 to 1, with values closer to 1 indicating better model fit.) = 0.953 > 0.95, Normed Fit Index (NFI, it is a normalized fit index, ranging from 0 to 1, with values closer to 1 indicating better fit.) = 0.934 > 0.9, indicating a good model fit [26].

## Discussion

The research findings indicate that the prevalence of myopia among primary and secondary school students is 27.3%, which is lower than the national average, likely due to the region’s emphasis on visual protection education and active prevention and control efforts. The Ningxia Hui Autonomous Region is a multi-ethnic area predominantly inhabited by the Hui people. Specific dietary and lifestyle habits inherent in Hui culture may indirectly influence the visual health of children and adolescents. Furthermore, the multicultural environment could impact the daily habits, educational methods, and family environments of young individuals, potentially affecting their visual status. Additionally, the region’ s predominantly plateau and mountainous terrain may facilitate diverse outdoor activities among adolescents, thereby enhancing their physical activity levels. Moreover, the relatively underdeveloped economic status of the region, coupled with the less widespread availability of electronic devices, may also contribute to the comparatively better visual health of its youth. However, preventing and treating myopia is a long-term endeavor, and a better understanding of the root causes of myopia among primary and secondary school students is crucial for promoting high-quality development of myopia prevention and control efforts in the region. This study found that the level of physical activity plays a masking effect between lifestyle behaviors and visual conditions, while academic stress moderates the relationship between lifestyle behaviors, physical activity level, and visual conditions.

Lifestyle behaviors encompass four dimensions: sleep behavior, dietary behavior, exercise behavior, and eye usage behavior, all of which are significantly negatively correlated with myopia. Prolonged near-distance eye usage is the primary factor influencing students’ vision status [27–28]. Adequate sleep, regular dietary habits, and balanced nutritional supplementation are all beneficial for preventing myopia [29–30]. The mean score of lifestyle behaviors among the group with visual impairment (44.78) is lower than that of the group with normal vision (47.86), indicating poorer lifestyle behaviors among the visually impaired group.

Physical activity level is significantly negatively correlated with lifestyle behaviors and myopia. The difference between the masking effect and the mediating effect is that after controlling for the mediating variable, the effect of the independent variable on the dependent variable will decrease, whereas controlling for the masking variable can increase the effect of the independent variable on the dependent variable. The masking effect of physical activity level enhances the impact of lifestyle behaviors on myopia. Physical exercise can promote blood circulation and the transport of nutrients, children and adolescents are in a critical stage of growth and development. The eyes require adequate nutrition for their development. Engaging in physical exercise can promote blood circulation, which is beneficial for transporting nutrients to all parts of the body, including the eyes. This helps in promoting the normal development of the optic nerve, eye muscles, and the eyeball, effectively preventing myopia [31–34]. Research suggests that children and adolescents with visual impairment typically engage in mild physical activity, with their physical activity level significantly lower than those of individuals with normal vision [35–37]. In this study, 68% of students in the visually impaired group engaged in low-intensity physical activity, and 29.1%, 26.6%, and 22.3% of students in this group were myopic when engaging in low, moderate, and high-intensity physical activity, respectively, indicating that the physical activity level of the visually impaired group is lower than that of the normal vision group. Therefore, we can infer that high-intensity physical activity can effectively suppress the onset of myopia, meaning that in the process by which lifestyle behaviors affect myopia, an increase in physical activity can further reduce the incidence of myopia.

Academic stress is significantly negatively correlated with lifestyle behaviors and physical activity level, and it moderates the process by which lifestyle behaviors influence physical activity level and visual conditions. Increased academic stress directly affects lifestyle behaviors, leading to a decrease in visual function regulation ability and potentially causing sleep disorders, which indirectly contribute to the occurrence of myopia [38–41]. Academic stress also reduces students’ participation in physical activities, thereby lowering their quality of lifestyle [42]. In this study, academic stress acts as a moderating variable, affecting lifestyle behaviors and physical activity level. On one hand, the mean values of lifestyle behaviors for students with no stress, a little stress, moderate stress, high stress, and extremely high stress are 48.99, 47.82, 46.80, 45.46, and 44.41, respectively. The scores for sleep behavior, eating behavior, and eye behavior also decrease as academic stress increases. It can be inferred that academic stress is a major factor influencing lifestyle behaviors and has a functional relationship. Increased academic stress leads to lower lifestyle behavior scores, indicating that controlling academic stress helps adolescents form good living habits. The mean academic stress for the group with poor vision is 2.96, higher than the 2.64 for the group with normal vision, suggesting that the impact of lifestyle behaviors on myopia decreases with the increase in academic stress. On the other hand, as academic stress increases, lifestyle behavior scores decrease, and physical activity levels also decrease, indicating that the influence of lifestyle behaviors on physical activity levels diminishes with the increase in academic stress. As a country with an exam-oriented education system, the increase in academic stress for students in China, due to the heavy workload, extracurricular tutoring classes, exam pressure, and the emphasis on academic achievement by educators, deprives students of physical activities, thereby reducing the potential benefits of physical activity in preventing myopia.

This study found that when students engage in moderate to high-intensity physical activity and experience mild to moderate academic stress, the prevalence of myopia is lower. Conversely, when their physical activity level is low and they face high to extremely high academic stress, the prevalence of myopia is higher. Resolving adolescent vision problems should focus on prevention and treatment, with an emphasis on prevention. This includes implementing regular screening mechanisms, improving adolescents’ lifestyle behaviors, encouraging their active participation in sports, and reducing academic stress. The ‘’Double Reduction’’ policy is a series of educational policies and measures implemented by the Chinese government to alleviate the extracurricular burden on primary and middle school students. ‘’Double Reduction’’ refers to reducing the burden of student homework and reducing the burden of off-campus training. Policy makers should continue to refine the ‘’Double Reduction’’ policy, while adopting more targeted strategies to address the diverse factors contributing to the pathogenesis. Schools should continue implementing the “Double Reduction” policy to lessen students’ academic burden and ensure adequate rest and physical activity. Additionally, a collaborative approach between families, schools, and communities is necessary. At the family level, parents should avoid placing excessive expectations and extra academic burdens on children, promote their active participation in sports, and foster a healthy mindset towards physical activity. At the school level, there should be increased emphasis on health education, including myopia prevention and control as an integral part. Schools should also provide students with more optional sports activities while reducing their academic burden, and abandon the educational concept of ‘’exam scores as the only criterion’’. Educators should shift their focus from a solely academic-oriented mindset to encourage student involvement in sports. Physical education teachers should adhere to the requirements of the “Compulsory Education Physical Education and Health Curriculum Standards (2022 Edition)” to teach students health knowledge and cultivate their physical abilities, healthy behaviors, and sports ethics. Physical activity is a crucial means to improve health and a significant safeguard against reducing myopia rates among adolescents. At the community level, proactive efforts should be made to conduct myopia awareness campaigns, engage eye care institutions, provide vision screening services, organize eye health activities, establish health records, and enhance awareness and knowledge of adolescent eye health.

### Limitations of the study

There were three main limitations to this study. First, the study was limited to the Ningxia Hui Autonomous Region, which may limit the generalizability of the findings. The environment, educational resources, and living habits in different regions may have different effects on students’ vision status. Second, the measurement tool was mainly in the form of a questionnaire and relied more on participants’ self-reports, which may lead to subjective bias and recall bias. In addition, this study employs the snapshot approach. Since this method collects data at a single time point, it cannot directly observe the dynamic process of causality, and it may also be influenced by unmeasured confounding factors. Because of the inability of cross-sectional data to accurately confirm causal pathways, subsequent studies can further clarify the causal mechanism through longitudinal or experimental research. In the future, consideration should be given to recruiting participants from different regions and on a large scale, and further analyzing the relationships among variables to ensure the generalizability of the results. In addition, there is a need to further incorporate objective measurement tools to increase the objectivity of the data. Further prospective or retrospective longitudinal studies can be conducted to validate these findings and explore potential causal relationships among the variables.

## Conclusion

The myopia prevalence among primary and secondary school students in the Ningxia Hui Autonomous Region is lower than the national average. Their myopia prevalence is related to their lifestyle behaviors, physical activity level, and academic stress. The established model reveals that the influence of lifestyle behaviors on visual health is controlled by physical activity level and academic stress. Improving lifestyle behaviors and reducing academic stress can effectively lower the myopia prevalence. physical activity level, as a confounding variable, significantly reduce myopia prevalence when effectively controlled. academic stress, as a moderating variable, affects lifestyle behaviors and physical activity level, thereby influencing visual health. Primary and secondary schools should deeply implement the “dual reduction” policy, adopt a collaborative approach between families, schools, and communities, and effectively carry out myopia prevention and treatment efforts.

## Data Availability

Our data were obtained in collaboration with a group from the School of Physical Education and Health at East China Normal University, and our data will be followed by other analyses, which we are unable to provide for the sake of data privacy. We have put the descriptive statistics of the data and related results of the analysis are placed in the main text, if you have questions about the data we will actively cooperate, we guarantee that our data is true and reliable.
